# Decompression of Subdural Hematomas Using an Intraosseous Needle in the Emergency Department: A Case Series

**DOI:** 10.5811/cpcem.2020.6.46069

**Published:** 2020-07-14

**Authors:** Brett Barro, Scott Kobner, Ashkon Ansari

**Affiliations:** * LAC+USC Medical Center, Department of Emergency Medicine, Los Angeles, California; † Keck School of Medicine of USC, Department of Emergency Medicine, Los Angeles, California; ‡ Antelope Valley Hospital, Department of Emergency Medicine, Lancaster, California

**Keywords:** emergency medicine, extra-axial hematoma, burr hole

## Abstract

**Introduction:**

Traumatic subdural hematomas beget significant morbidity and mortality if not rapidly decompressed. This presents a unique challenge to the emergency physician without immediate neurosurgical support.

**Case Report:**

We report two cases of patients in Los Angeles County with traumatic subdural hematomas and clinical deterioration in the emergency department (ED) who were treated with decompression using an intraosseous needle drill.

**Discussion:**

We believe these cases represent the first use of this technique to temporize a subdural hematoma in the ED.

## INTRODUCTION

Extra-axial hematomas (EAH), typically subdural (SDH) and epidural hematomas (EDH), are collections of blood surrounding the brain between the skull and various layers of meninges. Both entities represent neurosurgical emergencies for which surgical decompression, if indicated, is required to prevent secondary brain injury.^1^–^4^ Unfortunately, the time to definitive care by a neurosurgeon may be delayed due to various barriers such as hospital transfer times.

In addition to medical management, temporizing emergent trephinations (ie, burr holes) have long been performed by neurosurgeons in an attempt to decompress the intracranial space before taking patients to the operating room (OR). In the event of a delay, non-neurosurgeons have also performed this procedure successfully with favorable outcomes.^5^ This has most recently been reported for EDH using an intraosseous needle in the emergency department (ED).^6^ We present two separate cases of SDH evacuation with the use of the intraosseous needle (IO) by emergency physicians.

## CASE SERIES

### Case Report 1

A 65-year-old man with no known past medical history presented to a Level 1 trauma center after sustaining severe blunt head trauma. The patient was agitated with a Glasgow Coma Scale (GCS) of 11 (Eye(E): 3; Verbal(V): 3; Motor(M): 5). His physical exam revealed trauma to the left temporal area including palpable crepitus, a large hematoma, and bloody discharge from the left ear. Focused assessment with sonography in trauma (FAST) was negative for intraperitoneal and pericardial fluid. Plain films of his chest and pelvis were negative for acute injuries.

While being prepared for transport for computed tomography (CT), the patient became profoundly bradycardic and was treated for a suspected increase in intracranial pressure (ICP) with standard neuroprotective measures including elevation of his head, hypertonic saline, and mechanical hyperventilation. Despite these maneuvers, the patient went into cardiac arrest without a shockable rhythm. He was transfused two units type O positive blood and given epinephrine, sodium bicarbonate, calcium chloride, and tranexamic acid. Repeat FAST exam revealed no intraperitoneal or pericardial fluid.

After eight minutes of cardiopulmonary resuscitation (CPR), the decision was made to attempt decompression of a suspected EAH. An 11-blade scalpel was used to make a vertical incision three centimeters (cm) anterior and two cm superior to the left tragus over the temporal scalp where the culprit hematoma was thought to be located. A hemostat was then used to perform a subgaleal dissection. A 45-millimeter (mm) EZ-IO needle (Teleflex, Morrisville, NC) was subsequently inserted through the incision into the cranium. Using a syringe, roughly 10 milliliters (mL) of dark blood was evacuated from the extra-axial space with sudden return of spontaneous circulation.

Despite these efforts, the patient became pulseless again five minutes later while being stabilized for CT. CPR was resumed, and further attempts to evacuate blood through the IO needle were unsuccessful. CPR was eventually terminated. Post-mortem evaluation by the coroner confirmed the location of the IO needle in a subdural hematoma and verified the needle did not violate brain parenchyma. Unfortunately, the patient had concomitant subarachnoid hemorrhage with severe hydrocephalus leading to tonsillar herniation.

### Case Report 2

A 30-year-old man with no past medical history presented to a community hospital after sustaining significant blunt head trauma. Diagnostic imaging revealed a 16-mm, left-sided SDH and trace subarachnoid hemorrhage without evidence of herniation. No other clinically significant injuries were identified. Standard neuroprotective measures were undertaken and neurosurgery was consulted immediately.

Approximately 30 minutes after his initial imaging, the patient became bradycardic with a heart rate of 34 beats per minute (bpm) and hypertensive to 186/109 millimeters of mercury. The patient’s GCS deteriorated from 13 (E4V4M5) to 4T (E1V1TM2) without sedation or a long-acting paralytic. Hypertonic saline and mannitol were administered intravenously. Repeat imaging demonstrated an increase in SDH size to 20 mm, with new evidence of cisternal effacement, 12 mm of midline shift, and herniation. Neurosurgery was notified and the decision was made to transfer the patient to the OR for emergent craniotomy. Unfortunately, given the home-to-hospital commute time for the consultant, the soonest the patient could undergo surgery was over 30 minutes. After discussion with the neurosurgeon, the decision was made to attempt decompression with an EZ-IO.

Using CT guidance, the location of maximal clot depth was identified. Similar to the previous case, the IO needle was inserted into the extra-axial space. Using a three-milliliter (mL) syringe, roughly 15 mL of dark blood was evacuated and the patient’s heart rate increased from 30 bpm to 70 bpm. He was taken to the OR for craniotomy approximately one hour after the suspected herniation. Unfortunately, during his hospital stay, he did not have improvement in his neurologic status. Tracheostomy and gastrostomy tube placement were performed, and the patient was transitioned to a skilled nursing facility.

**CPC-EM Capsule**

What do we already know about this clinical entity?

Expanding subdural hematomas, if not decompressed in a timely fashion, often progress to brain herniation and irreversible neurological damage and death.

What makes this presentation of disease reportable?

The first two known cases of emergent decompression of subdural hematomas causing herniation utilizing an EZ-IO performed by emergency physicians without neurosurgical assistance.

What is the major learning point?

In austere environments, emergency physicians are capable of using EZ-IO needles to perform emergent trephinations and decompress extra-axial intracranial hemorrhages.

How might this improve emergency medicine practice?

As a heroic measure, emergency physicians can utilize this technique to temporize patients who may have impending herniation in order to get them to definitive neurosurgical care.

## DISCUSSION

It has previously been reported that a delay as short as 70 minutes from onset of anisocoria or coma with EAH portends a poor neurological outcome.^7^ Unfortunately, standard medical therapy for increases in ICP rapidly reach their limits of effectiveness. In such cases where herniation is inevitable, there is growing interest in the utility of emergent trephination after exhaustive medical treatment and prior to transfer to definitive surgery. This is of particular importance in the ED, where a large number of patients die from herniation syndromes after presenting with a neurologically intact exam, suggesting little-to-no primary brain insult.

Unfortunately, not all patients with an EAH and evidence of herniation present to a medical facility that has a neurosurgeon available to perform operative decompression in an acceptable time frame. In contrast to EDHs where burr holes may be a sufficient intervention, SDHs tend to have a large clot burden and persistent bleeding requiring a craniotomy or bone flap. It is, therefore, imperative that any ED trephination not delay transfer to the OR. However, as suggested by the Monro-Kellie doctrine, even small evacuations of blood can lead to dramatic decreases in ICP, reducing the risk of herniation while the patient awaits definitive care. There have been several case series where patients with an EAH and evidence of herniation were found to have improvement in Glasgow outcome scores after undergoing skull trephination by non-neurosurgeons prior to transfer.^8^,^9^ Although study results are inconsistent, it is difficult to dismiss a relatively simple procedure that potentially improves chances for favorable neurological recovery.^10^,^11^

The decision to perform an emergent trephination requires great deliberation. Required tools are not always available, the clinical scenario arises infrequently and, historically, this procedure is considered outside the scope of practice for generalists. This makes it challenging for emergency physicians to maintain a level of competency with the procedure or become credentialed. IO needles are readily available in most EDs and are a tool that all emergency physicians are proficient with, obviating the need to learn traditional trephination techniques. In addition, use of the IO theoretically reduces the risk of damaging the parenchyma as needle size can be chosen based on hematoma diameter measured on CT. Even with traditional trephination, non-neurosurgeons have similarly low rates of complications compared to neurosurgeons.^5^ It therefore seems prudent for emergency physicians to become familiar with this potentially life-saving procedure, particularly when working in austere environments. However, this intervention ought to be considered a heroic measure, only to be performed in circumstances when other life-saving interventions are not immediately available.

Recently, there was a report of non-neurosurgeons using an IO needle for decompression of an EDH as a temporizing measure in the United Kingdom.^6^ This technique was previously shown to be effective when used by neurosurgeons in a patient herniating from an EDH while awaiting operating room availability.^12^ We demonstrate that the EZ-IO can also be employed for relatively successful decompression of an acute SDH by non-neurosurgeons.

The described procedure took approximately 10 minutes to complete, which is consistent with previous reports.^6^,^7^ This amount of time should not cause any delay in transfer to definitive care and can be performed while transport is being arranged. We also advocate for the use of CT guidance and neurosurgical consultation before performing this procedure as done in *Case 2.* This involves placing a landmark (i.e., electrode sticker) on scalp prior to CT and measuring the distance to the center of the hematoma radiographically from this landmark (Image). Unfortunately, the patient in *Case 1* went into cardiac arrest before imaging. In this extreme circumstance, the decision was made to perform a landmark-based evacuation ipsilateral to the cranial trauma as a life-saving attempt. Before CT scanners became ubiquitous, emergent exploratory craniotomy for EAH was often performed using anatomical landmarks alone.^13^

Neither of the patients described survived to meaningful neurological recovery. This is the unfortunate outcome of most instances of brain herniation. Indeed, patients with EAH who arrive with GCS less than 6 or in cardiac arrest have minimal chance of meaningful recovery. However, a clinical improvement was initially established with emergent IO decompression. As emergency providers, we are tasked with making invasive intervention decisions that carry a low probability of conferring a positive morbidity or mortality outcome for patients. In the face of almost certain death, imparting a chance at survival should be weighed carefully.

## CONCLUSION

Emergent trephination in the appropriate clinical situation remains an active area of research. When approved by a neurosurgeon, the EZ-IO needle has been proposed as a useful tool to temporize well-selected patients with CT-confirmed EAH and clinical decompensation, when definitive care is delayed. We stress that emergency physicians should only consider employing this procedure in conjunction with neurosurgical consultation and in resource-limited settings so long as it does not delay transfer to the OR.

## Figures and Tables

**Image:**
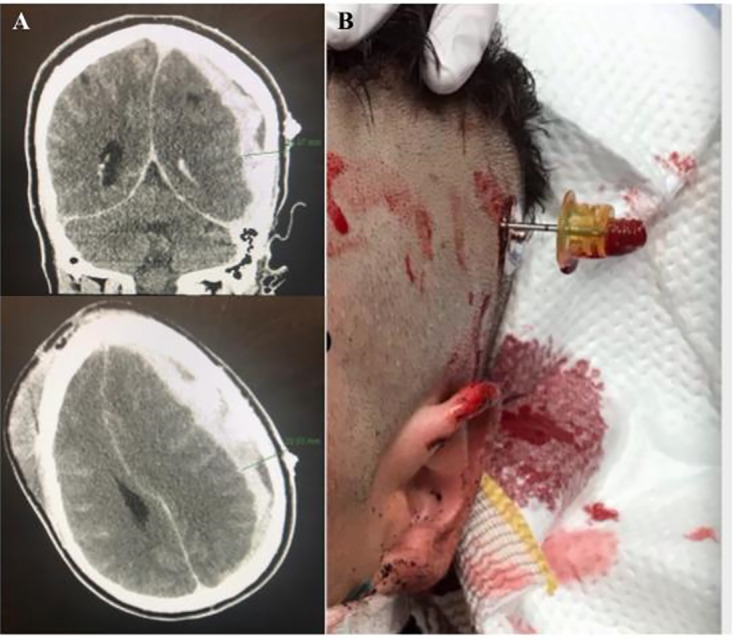
Intraosseous needle positioned on scalp (B) placed just inferior and anterior to marker seen on computed tomography (A) with maximal depth of hematoma measured to guide advancement of needle.
